# The Relationship Between Future Time Perspective and Psychological Violence Among Chinese College Students

**DOI:** 10.3389/fpsyg.2021.585837

**Published:** 2021-02-03

**Authors:** Kuiyun Zhi, Jian Yang, Yongjin Chen, Niyazi Akebaijiang, Meimei Liu, Xiaofei Yang, Shurui Zhang

**Affiliations:** ^1^School of Public Policy and Administration, Chongqing University, Chongqing, China; ^2^Aksu Vocational and Technical College, Aksu City, China

**Keywords:** future time perspective, psychological violence, future development, college students, China

## Abstract

Based on early experiences and current conditions, a future time perspective influences college students’ behaviors, while psychological violence critically threatens college students’ health. This study explored the relationship between a future time perspective and the psychological violence of perpetrators based on an online investigation of 1424 college students (87.1% women) aged 17 to 31 in China. The results showed that a future time perspective is significantly positively associated with psychological violence. Positive future orientation is negatively associated with psychological violence. Negative and confused future orientations are positively associated with psychological violence. These findings support the need to introduce an intervention regarding a future time perspective to reduce psychological violence among college students.

## Introduction

College students are in a period of life in which it is essential for them to think about many issues, such as their attitude toward life (Rice et al., 2012), lifestyle (Wang et al., 2015; Olfert et al., 2020), survival value (Lattie et al., 2019; Peacock, 2020), and social responsibility (Park et al., 2018; Jin et al., 2019). Life experiences show that positive feelings about the future will affect an individual’s educational and occupational achievement and even lifelong happiness (Tanaka, 2018). However, the significant cognitive and emotional changes that occur in college were suggested to increase problem behaviors, including psychological violence (Huang et al., 2012), making students feel negative or puzzled about the future. Psychological violence mainly refers to violence from perpetrators to victims that does not involve physical contact (McGee and Wolfe, 1991), which includes many forms, ranging from face-to-face confrontation such as insulting others verbally, to non-face-to-face confrontation, such as spreading rumors online, and occurs at the individual and group levels (Li et al., 2019). Zhi et al. (2013) investigated 4215 adolescent students aged 12 to 20 and summarized that psychological violence included pressure buildup, interpersonal attacks, verbal aggression and network violence. Largely caused by past bad experiences and current poor conditions (Xiang and Han, 2019; Cprek et al., 2020; Gan and Tang, 2020), psychological violence is one of the most prevalent forms of violent behaviors at college (Larranaga and Figueroa, 2011), which will influence college students’ future development (Fontaine and Réveillère, 2004; Shen, 2013; Fry et al., 2016). First, compared with obvious physical violent behaviors, psychological violence is usually more hidden, which makes it easier to ignore its long-term negative consequences, such as depressive states (Chen et al., 2011), posttraumatic stress disorder symptoms (Shen, 2013), poor educational performance (Tourigny et al., 2008; Fry et al., 2016; Han et al., 2017), and psychological traumatic expressions (Fontaine and Réveillère, 2004). Second, psychological violence exposure may account for revenge goal setting, which harms both victims and perpetrators in the future (Kunst, 2011; Jaggi and Kliewer, 2016). The above analysis implies that interventions in and the prevention of psychological violence among college students are becoming increasingly more urgent.

Extensive studies have been conducted to understand the risk factors for psychological violence. For instance, poor interpersonal relationships (Han et al., 2017), poor academic performance and problem behaviors (Kowalski and Limber, 2013; Bala et al., 2018), and early negative experiences, such as community violence exposure and harsh parenting in childhood (Sypher et al., 2019), may explain the high rates of psychological school bullying. Bandura’s (1997) self-efficacy theory suggests that early experiences, current appraisals, and future orientations influence current behaviors (Zimbardo and Boyd, 1999). Furthermore, future orientation, which is derived from reflections on early experiences and evaluations of current conditions (Lennings et al., 1998), directly influences current behaviors, including psychological violence, by shaping future goal setting (Markus and Nurius, 1986). Early experiences, such as community violence exposure and harsh parenting in childhood, were shown to promote negative future feelings, making adolescents more likely to engage in violence in the future (Sypher et al., 2019). Current interpersonal social network problems (Han et al., 2017), terrible study performance and bad habits such as smoking (Kowalski and Limber, 2013; Bala et al., 2018) reflect feelings of confusion about goals, which may explain the high rate of school bullying, including interpersonal attacks and verbal insults. There has been evidence suggesting a relationship between negative future orientation and the risk of verbal aggression, both offline and online (Kowalski and Limber, 2013; Li et al., 2019). The future time perspective (FTP) involves people’s thoughts, feelings, and actions related to their futures (Lyu and Huang, 2016). Although the evidence above implies a link between feelings about future events and psychological violence, very few studies have specifically examined the relationship between FTP and psychological violence.

Future time perspective could be applied to describe the way in which people feel about events in their future (Yang and Devaney, 2011). Different people may have different feelings about future events (Morselli, 2013). For example, previous studies measured college students’ future perspective in relation to work-related events (Sundberg et al., 1983; Cheng, 1997; Chen, 2006). Some students had positive perceptions, such as “finding an ideal job,” but some students had negative perceptions, such as “facing fierce employment competition.” Some students even felt confused, such as “being puzzled about career development.” Lyu and Huang (2016) explored the future feelings of college students and proposed that the feelings were based on a future time structure, which has been widely accepted in the Chinese context. According to Lyu and Huang’s (2016) study, FTP can be divided into three dimensions according to feelings: future positive, future negative and future confusion. Future positive describes a positive future orientation, which refers to generally positive, hopeful and happy feelings about future events. Future negative describes a negative future orientation, which refers to negative, hopeless and pessimistic feelings about future events. Future confusion describes confused future orientation, which refers to confused feelings about future events.

The association between reactive and proactive aggression in school bullying suggests that victims of psychological violence may retaliate against the perpetrators (Sourander et al., 2010; Barlett, 2015; Runions et al., 2018). Another study further indicates that victims of violence could be more likely to set revenge goals, rendering them more likely to seek satisfaction by harming perpetrators in the future (Kunst, 2011; Jaggi and Kliewer, 2016). The above evidence suggests that psychological violence may introduce a risk for the perpetrator in the future. Kruger et al. (2018) indicated that positive future orientation was correlated with preferences for low-risk future outcomes. Thus, people with a positive future orientation may worry about the risk of someone seeking revenge on them in the future, which in return would make them seek to avoid committing psychological violence. However, according to the previous study by Lyu and Huang (2016), those who were negative or confused about the future felt that the future was doomed and characterized by uncertainty. As a result, these individuals may not make efforts to avoid potential risk, threatening their future benefits, or care about the risk of revenge when committing psychological violence. The above analysis inspired us to look for more evidence regarding the relationship between different future orientations and psychological violence.

### Positive Future Orientation and Psychological Violence

Positive feelings about future events may prevent people from doing something harmful (Apostolidis et al., 2006; Fjær and Tutenges, 2016; Labãr and þepordei, 2019; Buckley et al., 2020). The literature provides evidence supporting a negative relationship between a positive future orientation and psychological violence. First, verbal attacks and network violence are regarded as risk-taking behaviors (Fjær and Tutenges, 2016; Buckley et al., 2020). Highly future-positive adolescents are less likely to have risk-taking behaviors (Apostolidis et al., 2006; Labãr and þepordei, 2019). Second, warm parenting experiences will make adolescents positive about future events, rendering them less likely to abuse others psychologically (Miller-Graff et al., 2016). Third, college students with a positive future orientation are more likely to focus on academic achievement (Teahan, 1958; Bembenutty and Karabenick, 2004; Kauffman and Husman, 2004). Those who highly value academic achievement are less likely to engage in violence, such as insulting others verbally (Kowalski and Limber, 2013). Moreover, a study indicates that college students with a positive future orientation are less likely to engage in network violence and verbal aggression (Han et al., 2017). Thus, we hypothesize that a positive future orientation is negatively associated with psychological violence (H1a).

### Negative, Confused Future Orientations, and Psychological Violence

Negative or confused feelings about future events may cause people to indulge in them at the present time (Kowalski and Limber, 2013; Bala et al., 2018). A positive relationship among negative future orientation, confused future orientation and psychological violence has also been implied previously. First, violence exposure (Hong et al., 2019; Lee et al., 2020) and posttraumatic stress symptoms (Marshall et al., 2020) could made teens feel negative or confused about the future, which may further push them to invade others (Sypher et al., 2019). Second, negative and confused future orientations are positively associated with low self-esteem among college students (Lyu et al., 2019). Low self-esteem is a risk factor for violence (Voisin et al., 2020). High self-esteem is linked to low psychological violence (Donnellan et al., 2005; Bushman et al., 2009). In addition, students with a negative or confused future orientation engage in network violence and verbal aggression more frequently (Desmyter and De Raedt, 2012; Kowalski and Limber, 2013; Kozik et al., 2015; Li et al., 2019). Consequently, we hypothesize that a negative future orientation and future confusion are positively associated with psychological violence (H1b).

## Materials and Methods

### Procedure and Participants

We recruited students from two departments of a college in western China to complete a survey. The students were told they may participate in a survey about college students’ psychological violence. After they read the online consent form, they had the option to choose whether to participate in this survey. The participants were told that this survey would not reveal their personal information, participation was completely voluntary, and joining the survey would authorize researchers to use their data. If the participants were willing to participate in the survey, they were given a link to this survey. Online questionnaires were sent to them via a website developed by Tencent Company^1^. In this platform, survey data were only accessible to the researcher, which referred to the team members of this project. The participants were instructed to complete online questionnaires, including information on age, academic performance, gender and region (urban or rural); a modified FTP scale based on Lyu and Huang’s study in 2016; and a psychological violence scale, which was applied in a previous study (Zhi et al., 2013). This research was approved by the ethics committee of the affiliated hospital of Chongqing University. The ethics committee reviewed the study proposal, consent of the participants and the introduction of the principal investigator. It also supervised the conduct of this research. In total, 1,537 responses were received in our study. The surveys of participants who provided the same answers to almost all the questions, completed the questionnaire too quickly, did not answer more than five items, or provided the same answers alternately were regarded as invalid. The linear interpolation method was applied to provide missing values. Finally, 1,424 valid responses were obtained. Participants aged from 17 to 31 (average age of 20.1) were included in the study. A total of 37.9% of the participants were from urban regions (*n* = 884), and 62.1% were from rural regions (*n* = 540). A total of 87.1% of the participants were women (*n* = 1240), and 12.9% of the participants were men (*n* = 184). A total of 61.1% of the participants rated their academic performance as average or not ideal (*n* = 870), and 38.9% of the participants were satisfied with their academic performance (*n* = 554).

Harman’s single factor test was applied to examine the potential effects of common method bias. All the scale items were subjected to exploratory factor analysis to determine whether a single factor emerged. The results indicated three factors with four values greater than 1.0, and the first factor accounted for 39.07% of the accumulated contribution rate (<40%) (Podsakoff et al., 2003). Thus, common method bias was not a problem in this study.

### Measures

#### Dependent Variables

Psychological violence was measured with a campus violence scale developed in Chinese (Zhi et al., 2013). In a previous study by Zhi et al. (2013) psychological violence was composed of four dimensions: pressure buildup (three items, e.g., “I used to threaten others verbally and warn them not to tell others”); verbal aggression (three items, e.g., “I used to talk about other people’s privacy, weaknesses or flaws”), interpersonal attacks (six items, e.g., “I used to intentionally post notes or other content that hurt others”), and network violence (five items, “I used to deliberately disclose others’ personal privacy information online”). Responses were given on a 5-point Likert scale ranging from strongly disagree (1) to strongly agree (5). A higher score indicates a higher level of psychological violence. In Zhi et al.’s study, the Cronbach’s α coefficients of verbal aggression, interpersonal attacks, pressure buildup, network violence and total scale were 0.65, 0.78, 0.64, 0.77, and 0.86, respectively. In this study, based on the data we collected, the Cronbach’s α coefficients of verbal aggression, interpersonal attacks, pressure buildup, network violence and total scale were 0.79, 0.94, 0.91, 0.96, and 0.97, respectively. The confirmatory factor analysis fit indexes provided good evidence of the construct validity of the psychological violence scale (χ^2^/*df* = 4.65, CFI = 0.984, TLI = 0.981, SRMR = 0.051, RMSEA = 0.018).

#### Independent Variables

This study applied a modified FTP scale based on a previous study (Lyu and Huang, 2016). Given the importance of work in college students’ future daily life, we used future work-related events such as career development to measure feelings toward specific future events using a scale design (Sundberg et al., 1983; Cheng, 1997). The scale consisted of 16 items and three subscales, namely, future positive, future negative and future confusion. The future positive subscale included six items (e.g., “I feel hopeful when I think I can find a good job in the future”); the future negative subscale included seven items (e.g., “I feel sad when I think about the employment pressure after graduation”); and the future confusion subscale included three items (e.g., “I feel confused because I don’t know how to achieve my career goals”). Responses ranged from strongly disagree (1) to strongly agree (5). A higher score indicates a higher level of FTP. In Lyu and Huang’s study, the Cronbach’s α coefficients of future positive, future negative, future confusion and the total scale were 0.83, 0.88, 0.76, and 0.90, respectively. In this study, based on the data we collected, the Cronbach’s α coefficients of future positive, future negative, future confusion and the total scale were 0.91, 0.91, 0.86, and 0.91, respectively. The confirmatory factor analysis fit indexes provided good evidence for the construct validity of the FTP scale (χ^2^/*df* = 4.85, CFI = 0.980, TLI = 0.970, SRMR = 0.036, RMSEA = 0.049). The Chinese version of the scale is reported in Appendix A.

#### Control Variables

Given that gender, age, region and academic performance were reported to influence psychological violence (Han et al., 2017; Li et al., 2019), we controlled for these variables in the regression analysis. Age was the actual age, while region and gender were coded as dummy variables (0 = woman and 1 = man for gender; 0 = rural and 1 = urban for region). Academic performance was measured with a 5-point Likert scale (1 = pretty poor and 5 = pretty good). Higher scores indicate better academic performance.

### Data Analysis

Data were analyzed by Mplus7.4. First, we determined the correlation coefficients of the study variables. Then, path analysis was applied to examine relationships among the dimensions of FTP and psychological violence.

## Results

Table 1 shows the correlations between FTP and psychological violence. FTP was significantly positively and weakly associated with psychological violence. Positive future orientation was significantly negatively associated with psychological violence and four dimensions, namely, pressure buildup, interpersonal attacks, verbal aggression, and network aggression. Negative and confused future orientations were significantly positively associated with psychological violence and its four dimensions. Considering that the relationship between FTP and psychological violence was statistically significant, regression analyses were conducted.

**TABLE 1:** Pearson correlations of the study variables.

Variable	1	2	3	4	5	6	7	8	9
1. Future-positive									
2. Future-negative	0.26**	–							
3. Future-confusion	0.22**	0.70**	–						
4. FTP	0.64**	0.84**	0.84**	–					
5. Pressure buildup	–0.02	0.29**	0.33**	0.26**	–				
6. Interpersonal attacks	−0.08**	0.25**	0.28**	0.20**	0.82**	–			
7. Verbal aggression	−0.08**	0.24**	0.26**	0.18**	0.76**	0.91**	–		
8. Network violence	−0.11**	0.22**	0.23**	0.15**	0.72**	0.86**	0.89**	–	
9. Psychological violence	−0.08*	0.26**	0.30**	0.21**	0.88**	0.96**	0.96**	0.93**	–

***p < 0.001, **p < 0.01, *p < 0.05.

**FIGURE 1:**
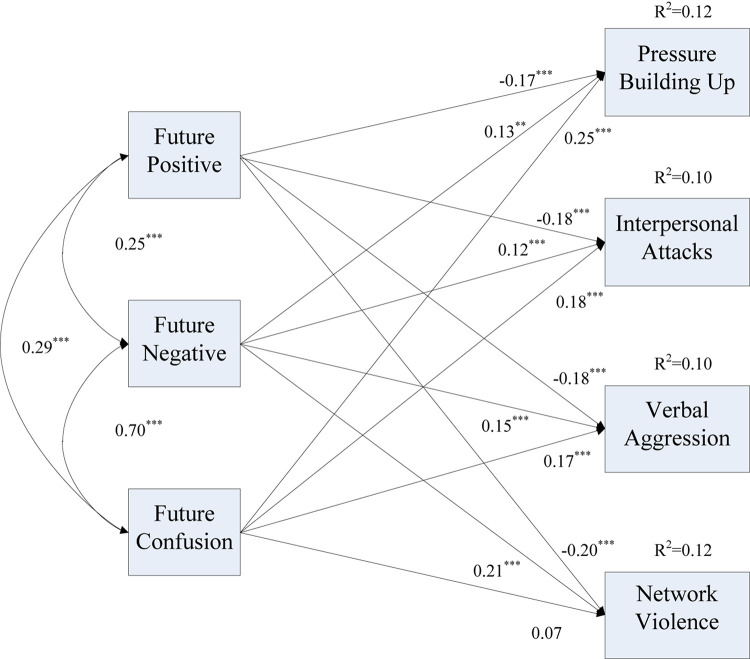
Path analysis of the sub-dimensions of FTP and psychological violence after adding the control variables. ^∗∗∗^*p* < 0.001, ^∗∗^*p* < 0.01, ^∗^*p* < 0.05.

Figure 1 shows the regression results of the dimensions of FTP and psychological violence. In this model, after controlling for participants’ age, gender, academic performance and region, the direct effects of FTP on pressure buildup, interpersonal attacks, verbal aggression, and network violence were 0.26, 0.16, 0.18, 0.17, and 0.09, respectively. The direct effect of future positive on psychological violence was −0.17. The direct effects of future negative and future confusion on psychological violence were 0.16 and 0.15, respectively. The figure illustrates that future positive negatively and significantly predicted all dimensions of psychological violence, including verbal aggression, interpersonal attacks, pressure buildup and network violence, while future negative had a significant positive effect on all dimensions of psychological violence. Moreover, future confusion was a positive and powerful predictor of verbal aggression, interpersonal attacks and pressure buildup. The direct effects of future confusion on pressure buildup, interpersonal attacks and verbal aggression are stronger than those of future negative except for network violence. Overall, both H1a and H1b were supported.

## Discussion

As described above, this study demonstrated the negative correlation between positive future orientation and psychological violence. This result was in accordance with previous studies, namely, positive future orientation predicts low risk-taking behavior (Apostolidis et al., 2006; Buckley et al., 2020), warm early experiences reduce psychological attacks (Miller-Graff et al., 2016), and pursuit of future positive targets prevents psychological insults (Kowalski and Limber, 2013). We proposed that goal-setting of the future time perspective could be applied to explain the relationship between positive future orientation and psychological violence (Chen, 2006). In general, college students are in a period during which their outlook on life and values are in the phase of formation to stabilization. They were likely to set positive future goals such as finding an ideal job and contributing to society, and they were more likely to experience positive feelings of such future goals. Positive goals will enable them to avoid factors that may threaten their future development (Runions et al., 2018) and make them more likely to consider others’ feelings about their actions. Thus, future positive college students are less likely to conduct psychological violence.

The positive relationship between negative, confused future orientations and psychological violence in this study was in accordance with previous findings that low self-esteem (Lyu et al., 2019), poor early experiences (Hong et al., 2019; Lee et al., 2020) and current poor condition (Marshall et al., 2020) may cause negative or confused feelings regarding future events, which may cause psychological violence to increase. Huang (1994) advocated that adolescents’ future cognition depends on experiences. Although most college students were more likely to perceive the positive content of future goals, some of them may not set positive future goals due to bad early experiences (Hong et al., 2019; Lee et al., 2020). Researches indicated that the lack of positive goals leads to college students’ problem behaviors (Chen, 2006). Thus, negative and confused future orientated college students were more likely to conduct psychological violence.

Meanwhile, previous studies on verbal aggression (Aloia and Solomon, 2015), interpersonal attack (Astrom et al., 2019; Yu et al., 2020) and network aggression (Chen et al., 2020; Sun et al., 2020; Zhang et al., 2020) agreed that negative and confused future orientations may predict psychological violence, but which orientation has stronger influence still remains uncertain. Data in this study suggest that a confused future orientation may have a stronger influence on pressure buildup, interpersonal attack and verbal aggression than negative future orientation. The present study suggests that compared with high future negative college students, high future confusion college students were more likely to pursue positive future goals (Huang et al., 1988; Huang and Yang, 1998; Chen, 2006; Lyu and Huang, 2016). However, as mentioned in the study by Lyu and Huang, high future confusion students possibly lack future planning, rendering them more likely to experience the pain of failure. Thus, they may be more likely to experience their negative feelings via committing psychological violence. For instance, for high future confusion college students, experiencing academic failure could result in psychological violence (Kowalski and Limber, 2013). Thus, a confused future orientation may be a more important risk factor for verbal aggression, interpersonal attack and pressure buildup than a negative future orientation.

We found that those who were positive about future events were less likely to engage in psychological violence, which may help to prevent psychological violence. For example, previous studies have proposed FTP interventions to manage adolescents’ aggression (Stolarski et al., 2016; Mizuta et al., 2018; Chadee et al., 2019; Orkibi and Ronen, 2019). Thus, we may consider introducing a future time perspective intervention to reduce psychological violence among college students.

However, we admitted that the major limitation of this study was the lack of evidence supporting the causal relationship between FTP and psychological violence. Although we regarded psychological violence as the dependent variable and FTP as the independent variable in path analysis. The study did not support the causal relationship between FTP and psychological violence, such as whether high psychological violence may promote a negative or confused future orientation, which was in contrast to our H1b. We mentioned previously that studies based on college students in China implied that victims of psychological violence may retaliate against their perpetrators (Sourander et al., 2010; Barlett, 2015; Runions et al., 2018), potentially putting the perpetrators at risk of psychological violence exposure. Meanwhile, psychological violence exposure could result in negative and externalizing behaviors, such as feeing puzzled about future development and giving up school work (Fry et al., 2016). Community psychological violence exposure was also suggested to trigger confusion about the future (Sypher et al., 2019). The findings above imply that psychological violence may predict a negative or confused future orientation. This study focused on the risk factors for psychological violence rather than its future consequences, so we did not further discuss the potential influence of psychological violence on negative/confused future orientation. Meanwhile, the cross-sectional study did not examine the causal relationship between negative/confused future orientation and psychological violence. Future studies that obtain longitudinal data to analyze the causal relationship between FTP and psychological violence would be valuable.

This study also has limitations in several other aspects. First, our data were collected from a college in western China via self-report online questionnaires. Meanwhile, an imbalance in gender existed, as there were significantly more female participants than male participants. Furthermore, the R square of the tested model was not very high. These limitations may challenge the robustness of this study. Thus, future studies should include samples with balanced participant characteristics and additional data sources (e.g., students from different schools) to validate the results. Second, we used college students to explore the relationship between FTP and psychological violence. However, psychological violence may occur in many contexts, such as the work place and home (Pai and Lee, 2011; Borah et al., 2017). The relationship between FTP and psychological violence in other contexts requires further examination. Finally, introducing mediators or moderators into the model of this study may be beneficial for explaining the association between FTP and psychological violence. Future studies should provide more evidence to examine the related mechanism.

## Data Availability Statement

The raw data supporting the conclusions of this article will be made available by the authors, without undue reservation.

## Ethics Statement

The studies involving human participants were reviewed and approved by the School of Public Policy and Administration, Chongqing University. Written informed consent from the participants’ legal guardian/next of kin was not required to participate in this study in accordance with the National Legislation and the Institutional Requirements.

## Author Contributions

KZ contributed to conceptualization, design, significant manuscript revisions and funding. JY contributed to the design, theory development, data analysis, and the final write-up. YC contributed to the conceptualization, design, and writing (original draft preparation). NA contributed to the data collection. ML contributed to the writing revision and modification. XY contributed to the literature search and SZ contributed to the modification. All authors contributed to the article and approved the submitted version.

## Conflict of Interest

The authors declare that the research was conducted in the absence of any commercial or financial relationships that could be construed as a potential conflict of interest.

## Footnotes

1 https://wj.qq.com/

## Appendix A

### Future Time Perspective Scale

1. I feel sad when I think about the employment pressure after graduation.

2. I feel worried when I think that I might not find the job I want in the future.

3. I feel anxious when thinking about the competition I will face regarding jobs.

4. I feel anxious when thinking about the great pressure of having a job in the future.

5. I feel sad that I will not be able to achieve my career goals in the next 10 years.

6. I feel frustrated when thinking that I might not be appreciated for years.

7. I feel annoyed when thinking that I might get tired of the job after working for years.

8. I feel hopeful when I think I can find a good job in the future.

9. I feel excited when thinking about the opportunity I’m facing after taking a job.

10. I feel warm when I think I’ll make new friends after taking a job.

11. I feel happy when I think that my career will develop very well.

12. I feel happy when I think about the sound development of my position.

13. I feel happy when thinking that I will be appreciated by my superiors in the future.

14. I feel dazed because I don’t know what kind of work pressure I’m going to face.

15. I feel confused when I think about the uncertainty about my future career.

16. I feel confused because I don’t know how to achieve my career goals.
